# Risk of Retinal Redetachment After Cataract Surgery Following Retinal Detachment Repair in Myopic and Highly Myopic Eyes

**DOI:** 10.1177/24741264261418517

**Published:** 2026-02-24

**Authors:** Yeabsira Mesfin, Leo Arnal, Anish Salvi, Karen M. Wai, Frank Brodie, Eubee Koo, Andrea L. Kossler, Euna Koo, Ehsan Rahimy, Prithvi Mruthyunjaya, Chase A. Ludwig

**Affiliations:** 1 School of Medicine, University of California, San Francisco School of Medicine, San Francisco, CA, USA; 2 Department of Ophthalmology, Byers Eye Institute, Stanford University School of Medicine, Palo Alto, CA, USA; 3 Department of Ophthalmology, Palo Alto Medical Foundation, Palo Alto, CA, USA

**Keywords:** retinal detachment, redetachment, cataracts, cataract surgery, myopia, vitrectomy

## Abstract

**Purpose:** To describe how risk factors such as repair of rhegmatogenous retinal detachment (RRD), cataract extraction, and myopia interrelate to influence the risk of retinal redetachment. **Methods:** This retrospective cohort study included patients with phakic RRD who had subsequent cataract extraction. The incidence and risk of redetachment were compared using Cox regression and χ^2^ analyses. Stratified analyses were performed based on time after cataract extraction, age, myopia status, and retinal repair type. **Results:** Of 1222 patients identified, no significant association was found between myopia and the incidence of redetachment, although the proportion of redetachments increased with the degree of myopia (nonmyopes, 8.5%, myopes, 9.5%, high myopes, 15.6%; *P* = .36). Myopia and high myopia were not associated with an increased risk of redetachment over time (hazard ratio, 1.01, *P* = .96; hazard ratio, 1.54, *P* = .35, respectively). Additionally, the incidence of redetachment was not significantly correlated with the time after cataract extraction (*P* = .33). A significant difference was observed between the incidence of redetachment and age (*P* = .003). Patients between 18 and 35 years experienced the highest incidence of redetachment within 1, 3, and 12 months after cataract extraction (5.26%, 7.02%, 7.02%, respectively). Such patients were overrepresented among those who underwent complex surgeries for initial phakic RRD repair (30-39 years, residual: 2.71; 40-49 years, residual: 3.32). **Conclusions:** Among patients with a phakic RRD, myopia did not significantly increase the risk of redetachment after cataract extraction. However, an upward trend was noted between the proportion of redetachments and the degree of myopia. Younger patients exhibited the highest incidence of redetachment and should be closely monitored after cataract extraction.

## Introduction

Many patients undergoing rhegmatogenous retinal detachment (RRD) repair are either myopic or highly myopic, and the majority will develop cataracts that require extraction after RRD surgery.^1,2^ Myopia is a significant risk factor for RRD and recurrent RRD.^3,4^ Anatomically, in axial myopia, the retina and vitreous are mechanically stretched, leading to liquefaction of the vitreous at a younger age and increased vitreoretinal traction.^5^ Therefore, younger myopic patients are more prone to early-onset posterior vitreous detachments (PVDs) and early-onset RRDs. Among these patients, such detachments can occur in the absence of PVDs and are commonly attributed to retinal dialysis or round retinal holes.^6^ Additionally, surgical repair of these RRDs, particularly with pars plana vitrectomy (PPV), subsequently increases the postoperative risk of cataract formation. The use of intraocular tamponade agents such as sulfur hexafluoride and perfluoropropane, along with changes in oxygen distribution, predisposes the crystalline lens to nuclear sclerosis.^7–12^ These structural alterations explain why 60% of patients develop cataracts after tamponade with silicone oil and why up to 96% experience lenticular changes within 2 years of surgery.^13,14^

The risk of redetachment after RRD surgery varies by procedure type. Reported redetachment rates among phakic patients undergoing PPV, scleral buckle repair, combined scleral buckle/PPV, and pneumatic retinopexy are 26.8%, 31.0%, 19.0%, and 23.5%, respectively. However, a smaller subset of redetachments (2.8%, 5.3%, 1.8%, and 9.1%) occur within approximately 6 years after cataract surgery.^15,16^ Similarly, among pseudophakic patients, redetachment rates are estimated to be approximately 16% after PPV and 8% after scleral buckle/PPV procedures.^17^ Vitreous changes after the removal of a cataract can lead to vitreoretinal traction on any remaining vitreous and PVDs in eyes that underwent scleral buckle or pneumatic retinopexy.^18^ Despite cataract surgeries being the most frequently performed surgery in the world, however, the surgical outcomes for patients with a concurrent history of RRD are less clear.^19^

Given that both RRD and cataract surgeries are independently associated with increased risk of subsequent RRD, the question of how previous RRD impacts the risk of redetachment after surgery for the fated cataract after PPV is of particular interest.^10,20^ This raises a growing concern of the risk of postoperative redetachment among myopic patients with a history of RRD who require cataract surgery. This retrospective cohort study aimed to analyze the incidence of redetachment among nonmyopic, myopic, and highly myopic patients who had cataract surgery after previously having RRD repair. In doing so, we hope to support patient counseling and clinical decision-making by physicians by better classifying the risks associated with cataract surgery after RRD repair. We hypothesize that the risk of redetachment increases with the degree of myopia.

## Methods

This study adhered to the tenets of the Declaration of Helsinki. Data were analyzed from the TriNetX Health Research Network (Cambridge, MA). This database compiles anonymized electronic health records (EHRs) from over 124 million patients across 9 countries, including 60 healthcare organizations in the United States. TriNetX complies with Health Insurance Portability and Accountability Act regulations and holds International Organization for Standardization 27001:2013 certification. The study was exempt from institutional review board approval due to the use of anonymized patient data.

### Inclusion and Exclusion Criteria

Data for the study were accessed on April 10, 2024, from the TriNetX platform, which provides a comprehensive range of EHR information, such as diagnoses, treatments, prescriptions, laboratory results, genetic data, hospital admissions, surgeries, and outpatient visits. At the time of data collection, the database included records of 124 043 499 patients, covering the period from January 1, 2003, to April 10, 2024. The study adhered to the Strengthening the Reporting of Observational Studies in Epidemiology guidelines for reporting observational cohort studies.

**Figure 1.**
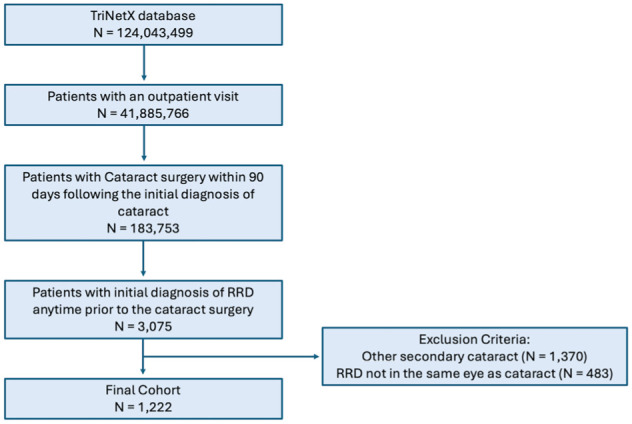
Flowchart showing how the study population was generated from the TriNetX database.

Among all the patients in the TriNetX, we identified those who had an outpatient visit (Current Procedural Terminology [CPT]: 99202, 99203, 99204, 99205, 92002, 92004, 99211, 99212, 99213, 99214, 99215, 92012, 92014, 99024) (Figure 1). Patients diagnosed with cataracts (International Classification of Diseases [ICD], 10th revision: H25) who underwent cataract surgery (CPT: 66982, 66984) within 90 days of their initial diagnosis and had a retinal detachment with retinal break (ICD-10: H33.0) before the cataract surgery were included. All patients with Soemmerring’s ring (ICD-10: H26.41) or other secondary cataracts (ICD-10: H26.49) were excluded, as well as patients younger than 18 years.

### Incidence of Redetachment by Patient Characteristics

This study analyzed the characteristics and factors associated with redetachment after cataract surgery. To ensure accurate matching between the laterality of cataract surgery and previous detachments, cases with bilateral cataract diagnoses were excluded. For redetachments occurring after cataract surgery, laterality was confirmed using ICD codes for retinal detachment. These codes were cross-referenced with CPT codes for repairs of retinal detachment with scleral buckling (CPT: 67107), vitrectomy (CPT: 67108), pneumatic retinopexy (CPT: 67110), and vitrectomy with membrane peeling (CPT: 67113) to verify a redetachment had occurred. In contrast to ICD codes, which may persist beyond the initial diagnosis, CPT codes correspond to specific surgical procedures and are not reused. This distinction minimized the risk of misclassifying persistent ICD codes as new instances of detachment.

Disease definitions relied on specific ICD codes (Figure 1). Categories of race included Asian, American Indian or Alaska Native, Black or African American, Native Hawaiian, White, unknown, or other, as sourced from TriNetX. Categories of ethnicity included Hispanic or Latino, non-Hispanic or Latino, and unknown. We also generated prevalence tables to characterize patients with retinas that remained attached vs patients with retinas that redetached, results by age (10-year bins from 18 to >90), sex, race, ethnicity, and myopia status (nonmyopes, myopes [ICD-10: H52.1*], and high myopes [ICD-10: H44.2*]). The average follow-up interval for each age group was also calculated, with an analysis of variance performed to compare follow-up times. We also used a χ^2^ analysis to compare the types of initial phakic retinal detachment repair surgery each age group received and examined standardized residuals to assess which age groups were overrepresented or underrepresented across each surgery type.

The incidence of redetachment after cataract surgery was stratified by myopia status to further explore the association of myopia with redetachment. The analysis examined the number of cases that occurred within different timelines: within 1 month after cataract surgery, between 1 and 3 months, between 3 months and 1 year, and any time after 1 year. A similar analysis was performed, stratifying the results by age bins determined by clinical significance (18-35, 35-55, 55-75, >75 years).

Additionally, we examined the number of surgeries required to treat redetachment after cataract surgery across myopia status (nonmyopes, myopes, high myopes) and stratified the results by the type of cataract surgery (complex or routine), the presence of PVD (ICD-10: H43.819) or proliferative vitreoretinopathy (PVR; ICD-10: H35.20), and the type of surgery performed to treat detachment (PPV [CPT: 67108], scleral buckle [CPT: 67107], scleral buckle/PPV or complex repair [CPT: 67113], pneumatic retinopexy [CPT: 67110]). When multiple surgeries (cataract or to treat detachment) were performed, the first surgery type among the patient’s record was selected to classify the type of surgery. A single-surgery success was assumed if there was only 1 occurrence of the CPT codes to treat detachment after the first cataract surgery, and we assumed that additional surgery was required if the CPT codes appeared more than once. Statistical differences between categorical variables (sex, race, ethnicity, myopic status, surgery type, etc) were assessed using the χ^2^ test, with a significance threshold of *P* < .05 using Python (libraries: dask, pandas, sklearn, lifelines).

### Survival Analysis for Redetachment

Finally, a time-to-event analysis was performed using a multivariate Cox regression to examine the association between myopia status and the risk of redetachment over time, with assumptions for proportional hazards. The model was adjusted for age, sex, race, and ethnicity, given how these variables have been established to influence detachment and refractive status.^21,22^ We also plotted Kaplan-Meier survival curves that showed the time from cataract surgery to redetachment, stratified by myopia classification. All statistical analyses were performed using Python (libraries: dask, pandas, sklearn, lifelines).

## Results

A total of 1222 patients were identified in the TriNetX database after applying our strict inclusion and exclusion criteria. Table 1 describes the baseline characteristics of patients who experienced retina redetachment vs those whose retinas remained attached. Overall, 792 patients (64.8%) were male, and 430 (35.2%) were female. Regarding race, 882 patients (72.2%) identified as White, 155 (12.7%) as Black or African American, 27 (2.2%) as Asian, 6 (0.5%) as Native Hawaiian, 4 (0.3%) as American Indian or Alaska Native, 40 (3.3%) as other, and 108 (8.8%) as unknown. Regarding ethnicity, 151 patients (12.7%) identified as Hispanic or Latino, 921 (75.4%) as non-Hispanic or Latino, and 150 (12.3%) as unknown. Finally, 1032 patients (84.5%) had no diagnosis of myopia, 158 (12.9%) were myopic, and 32 (2.6%) were highly myopic. In total, 31 patients (2.5%) underwent yttrium aluminum garnet capsulotomy. The *P*-values to assess statistical differences between the different categories were significant for age (*P* = .003) but not for sex (*P* = 1.00), race (*P* = .90), ethnicity (*P* = .19), myopia status (*P* = .36), or yttrium aluminum garnet capsulotomy (*P* = .26). Table 2 presents the standardized residuals of the observed vs the expected representation of each age group when our cohort was stratified by the type of initial phakic retinal detachment repair surgery. Complex repair surgeries were performed more frequently than expected among patients between ages 30 and 39 years (residual: 2.71) and 40 and 49 years (residual: 3.32) and less frequently among 50- to 59-year-olds (residual: −2.15). Patients between 40 and 49 years were found to be less likely to receive vitrectomies on initial retinal detachment repair (residual: −2.06). Supplemental Table 1 highlights the average follow-up interval for each age group. Patients between ages 30 and 39 and 40 and 49 years exhibited the shortest follow-up intervals of approximately 6.58 ± 6.75 and 6.97 ± 6.33 years, respectively. Patients between ages 70 and 79 years and 60 and 69 years were found to have the longest follow-up intervals of approximately 10.14 ± 9.39 years and 8.96 ± 8.20 years, respectively. A significant difference was observed when comparing the follow-up intervals across each age group (*P* < .05).

**Table 1.** Baseline Characteristics of Patients in TriNetX by Presence of Redetachment After Cataract Surgery.

Characteristics	Remained Attached (n = 1114)	Redetached (n = 108)	*P* Value
Age (y), n (%)		n (%)	
20-29	25 (2.3)	8 (7.4)	.003
30-39	73 (6.6)	15 (13.9)	
40-49	152 (13.6)	11 (10.2)	
50-59	414 (37.2)	30 (27.8)	
60-69	330 (29.6)	32 (29.6)	
70-79	102 (9.2)	10 (9.3)	
80-89	17 (1.5)	2 (1.9)	
≥90	0 (0.0)	0 (0.0)	
Sex, n (%)			
Male	722 (64.8)	70 (64.8)	1.00
Female	392 (35.2)	38 (35.2)	
Other	0 (0)	0 (0)	
Race, n (%)			
Native Hawaiian	6 (0.5)	0 (0.0)	.9
Asian	26 (2.3)	1 (0.9)	
Black	141 (12.7)	14 (13.0)	
American Indian or Alaska Native	4 (0.4)	0 (0.0)	
Other	37 (3.3)	3 (2.8)	
Unknown	97 (8.7)	11 (10.2)	
White	803 (72.1)	79 (73.2)	
Ethnicity, n (%)			
Hispanic or Latino	133 (11.9)	18 (16.7)	.19
Not Hispanic or Latino	840 (75.4)	81 (75.0)	
Unknown	141 (12.7)	9 (8.3)	
Myopia status, n (%)			
No myopia	944 (84.7)	88 (81.5)	.36
Myopia	143 (12.8)	15 (13.9)	
High/pathologic myopia	27 (2.4)	5 (4.6)	
Underwent yttrium aluminum garnet capsulotomy, n (%)	30 (2.7)	1 (0.9)	.26

**Table 2.** Types of Initial Phakic Retinal Detachment Repair Surgery by Age Group and the Respective Standardized Residuals Showing the Difference Between Expected vs Observed Frequencies for Each Surgery Type.

Age Group	Type of Initial Phakic Retinal Repair Surgery^a^				*P* Value
	Vitrectomy n (%)	Scleral Buckle n (%)	Pneumatic Retinopexy n (%)	Complex Repair n (%)	
20-29	13 (2.1)	5 (6.7)	1 (2.4)	11 (3.4)	< .05
30-39	26 (4.2)	8 (10.7)	1 (2.4)	30 (9.2)	
40-49	64 (10.3)	10 (13.3)	2 (4.8)	65 (19.8)	
50-59	249 (39.9)	25 (33.3)	18 (42.9)	96 (29.3)	
60-69	201 (32.2)	17 (22.7)	13 (31.0)	84 (25.6)	
70-79	61 (9.8)	9 (12.0)	6 (14.3)	34 (10.4)	
80-89	10 (1.6)	1 (1.3)	0 (0.0)	7 (2.1)	
Standardized Residuals					
20-29	−0.95	1.08	−0.08	0.97	
30-39	−1.8	0.77	−0.89	2.71	
40-49	−2.06	0.73	−1.43	3.32	
50-59	1.39	−0.41	0.62	−2.15	
60-69	1.19	−0.89	0.33	−1.51	
70-79	−0.36	0.35	0.94	0.03	
80-89	−0.2	−0.02	−0.83	0.63	

a These numbers reflect the patients from the TriNetX registry with available data regarding their initial surgery type.

Table 3 presents the incidence of redetachment after cataract surgery by myopia classification. Overall, redetachment was observed within 1 month in 21 cases (1.7%), within 3 months in 19 cases (1.6%), within 1 year in 35 cases (2.9%), and beyond 1 year in 33 cases (2.7%). The overall proportion of redetachment any time after cataract surgery increased with the severity of myopia. Among high myopes, 1 case (3.1%) experienced redetachment within 1 month, 1 case (3.1%) between 1 and 3 months, and 3 cases (9.4%) beyond 1 year. Compared with nonmyopes and high myopes, myopes experienced a higher proportion of redetachment within 1 year (8 cases, 5.1%). However, the trend was not statistically significant (*P* = .33).

**Table 3.** Incidence of Redetachment After Cataract Surgery by Myopia Classification.

Timeline	Patients, n (%)				*P* Value
	All (n = 1222)	Nonmyopes (n = 1032)	Myopes (n = 158)	High Myopes (n = 32)	
Within 1 month	21 (1.7)	19 (1.8)	2 (1.3)	0 (0.0)	.33
Between 1-3 months	19 (1.6)	15 (1.5)	3 (1.9)	1 (3.1)	
Within 1 year	35 (2.9)	26 (2.5)	8 (5.1)	1 (3.1)	
>1 year	33 (2.7)	28 (2.7)	2 (1.3)	3 (9.4)	

**Figure 2.**
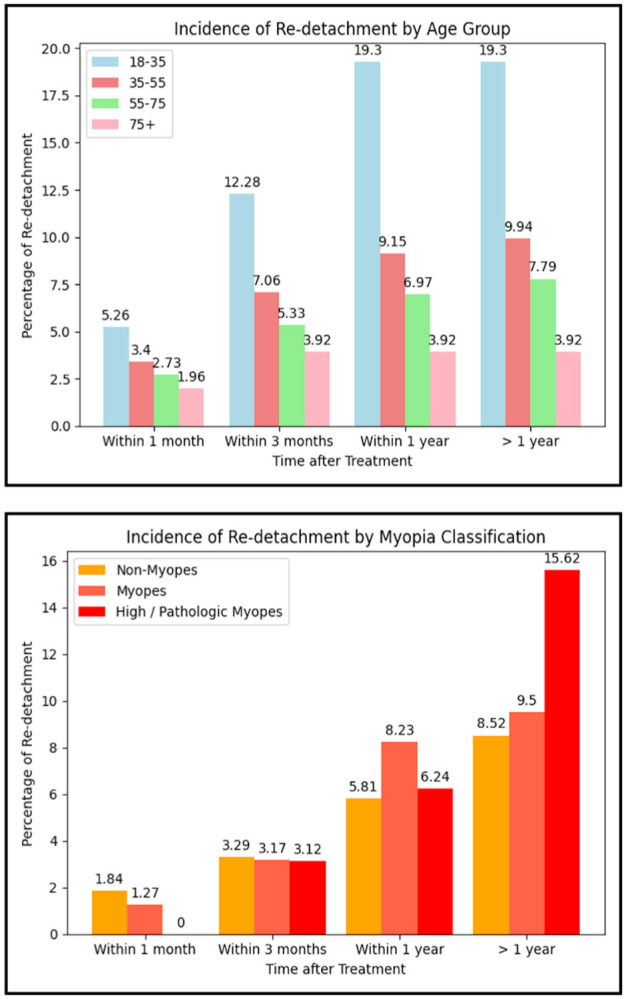
(Top) Incidence of redetachment by age. (Bottom) Myopia classification: No myopia, myopia, and high/pathologic myopia.

Figure 2 shows the incidence of redetachment after cataract surgery, broken down by myopia classification and age group. The 18- to 35-year age group experienced the highest rate of redetachment within 1 month (5.3%), followed by the 35- to 55-year age group (3.4%). This pattern persisted at the 3-month mark, with the 18- to 35-year age group again showing the highest incidence (7.0%). As age increased, the rate of redetachment consistently declined across all timeframes. Beyond 1 year, there were no recorded cases of redetachment in the 18- to 35-year and >75-year age groups.

Table 4 presents the single-surgery anatomic success rates for redetachment repair after cataract surgery, stratified by myopia status. Among the 88 nonmyopic patients, 64 (72.7%) achieved single-surgery success, while 24 (27.3%) required more than 1 procedure. When patients with single-surgery success were compared with patients requiring more than 1 surgery, the type of cataract surgery, either routine or complex, did not significantly differ (*P* = .31). Specifically, 43 patients (67.2%) who underwent routine surgery achieved single-surgery success, compared with 19 (79.2%) who required additional surgeries. No cases of PVR were recorded in patients requiring multiple surgeries, while 2 patients (3.1%) in the single-surgery success group had PVR (*P* = NA). Lastly, although differences in the type of redetachment (RRD) repair surgery were noted, statistical significance was not reached when comparing those with and without single-surgery success (*P* = .13).

**Table 4.** Single Surgery Anatomic Success for Redetachment Repair After Cataract Surgery.

	Nonmyopes (n = 88)				Myopes (n = 15)		High/Pathologic Myopes (n = 5)		
Variables	Single Surgery Success, n (%) 64 (72.7)	>1 Surgery Required, n (%) 24 (27.3)	*P* value	Single Surgery Success, n (%) 13 (86.7)	>1 Surgery Required, n (%) 2 (13.3)	*P* value	Single Surgery Success, n (%) 3 (60.0)	>1 Surgery Required, n (%) 2 (40.0)	*P* value
Cataract surgery type, n (%)									
Routine	43 (67.2)	19 (79.2)	.31	11 (84.6)	1 (50.0)	.37	2 (66.7)	1 (50.0)	1.00
Complex	21 (32.8)	5 (20.8)		2 (15.4)	1 (50.0)		1 (33.3)	1 (50.0)	
Clinical characteristics									
PVD present	5 (7.8)	0 (0.0)	NA	4 (30.8)	0 (0.0)	NA	0 (0.0)	0 (0.0)	NA
PVR present	2 (3.1)	0 (0.0)		0 (0.0)	0 (0.0)		0 (0.0)	0 (0.0)	
Surgery type									
Vitrectomy	32 (50.0)	14 (58.3)	.13	6 (46.2)	1 (50.0)	.35	1 (33.3)	0 (0.0)	NA
Scleral buckle	1 (1.6)	3 (12.5)		2 (15.4)	0 (0.0)		1 (33.3)	0 (0.0)	
Scleral buckle/PPV^a^	30 (46.9)	12 (50.0)		4 (30.8)	0 (0.0)		1 (33.3)	2 (100.0)	
Pneumatic retinopexy	4 (6.3)	0 (0.0)		1 (7.7)	1 (50.0)		0 (0.00%)	0 (0.0)	

a Includes both scleral buckle/PPV and complex repair surgeries.

Abbreviations: NA, not applicable; PPV, pars plana vitrectomy; PVD, posterior vitreous detachment; PVR, proliferative vitreoretinopathy.

In the myopic group, 13 patients (86.7%) achieved single-surgery success, while 2 (13.3%) required additional surgeries. The type of cataract surgery did not significantly differ between patients with and without single-surgery success (*P* = .37). Specifically, 11 patients (84.6%) with single-surgery success underwent routine cataract surgery, compared with 1 patient (50.0%) in the multiple-surgery group. Neither subgroup of myopic patients had PVR (*P* = NA). Six patients (46.2%) in the single-surgery success group had vitrectomies, 2 patients (15.4%) had scleral buckles, 4 (30.8%) had complex repairs, and 1 patient (7.7%) had pneumatic retinopexy. In contrast, 1 vitrectomy (50.0%) and 1 pneumatic retinopexy (50.0%) were performed in the multiple-surgery group. These differences were not statistically significant (*P* = .35).

In the high/pathologic myopia group, 3 patients (60.0%) achieved single-surgery success, while 2 (40.0%) required additional surgeries. Routine cataract surgery was performed in 2 patients (66.7%) in the single-surgery success group and 1 patient (50.0%) in the multiple-surgery group (*P* = 1.00). There were no cases of PVR in either group (*P* = NA). All RRD repair surgeries in the multiple-surgery group were complex repairs. In the single-surgery success group, 1 vitrectomy (33.3%), 1 scleral buckle (33.3%), and 1 complex repair (33.3%) were performed. These results were not statistically significant (*P* = NA).

**Figure 3.**
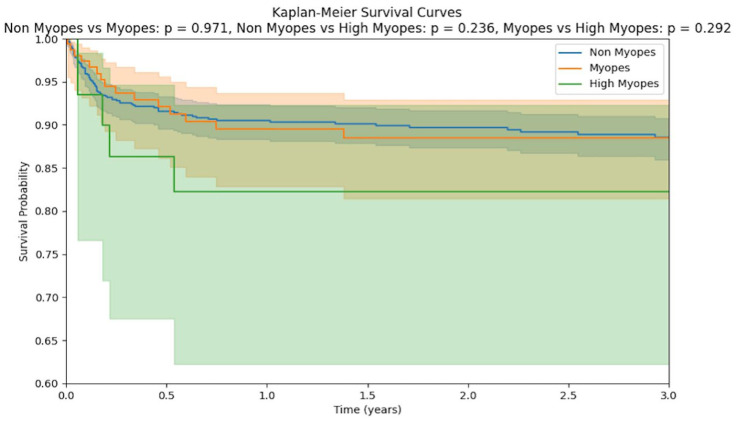
Survival curves showing time from cataract surgery to redetachment, stratified by myopia classification: Nonmyopes vs myopes, nonmyopes vs high myopes, and myopes vs high myopes.

Table 5 presents the hazard ratios for redetachment risk after cataract surgery, stratified by myopia level and adjusted for age, sex, race, and ethnicity. Compared with nonmyopes, myopic patients had a hazard ratio of 1.01 (*P* = .96), suggesting no significant difference in redetachment risk compared with nonmyopes. High myopes showed a hazard ratio of 1.54 (*P* = .35), indicating a possible trend toward increased risk, but this was not statistically significant. A Kaplan-Meier survival curve is shown in Figure 3 comparing survival rates between nonmyopes and myopes, nonmyopes and high myopes, and myopes and high myopes. Although high myopes were at a higher risk of redetachment compared with nonmyopes and myopes, the difference was not statistically significant (*P* = .24).

**Table 5.** Risk of Redetachment by Myopia Classification Compared With Nonmyopes.

Myopia Classification	HR (95% CI)	*P* Value
Nonmyopes (reference)	NA	NA
Myopes	1.01 (0.58-1.76)	.96
High myopes	1.54 (0.62-3.86)	.35

Abbreviations: HR, hazard ratio; NA, not applicable.

## Conclusions

This study investigated the risk of retinal redetachment in phakic patients who underwent retinal detachment repair and subsequently required cataract surgery. Overall, we found that myopic status did not significantly impact the risk of retinal redetachment after cataract surgery among patients with a history of phakic retinal detachment repair. However, a positive trend was observed between the proportion of detachments and the degree of myopia. The type of redetachment repair surgery was not found to influence the likelihood of primary anatomic success. Notably, a significant difference was identified between the incidence of redetachment and patient age, with the highest incidence of redetachment after cataract surgeries seen in younger patients. These results provide important insights into the interplay between patient characteristics and surgical outcomes in high-risk groups.

Myopia and cataract surgeries are both independently associated with an increased risk of retinal detachment.^3,23^ Our results demonstrated that, over time, the proportion of redetachments increases with the degree of myopia; however, contrary to our original hypothesis, the relationship between myopia and retinal redetachment was not statistically significant (*P* > .05), but an upward trend was observed. Although the largest proportion of pseudophakic redetachments in the beyond 1-year period was observed for patients with high myopia, accounting for approximately 9.4% of these patients, a statistically significant correlation was not observed. Our survival analysis supports these conclusions as well. A greater proportion of myopes and high myopes displayed redetachments over time compared with nonmyopes, but a significant difference was not observed in the survival rates of these subgroups when compared with one another. Overall, these results suggest that myopia may not be a large contributor toward redetachments, offering reassurance to myopic patients considering a cataract extraction.

A similar trend was observed by Ripandelli et al,^24^ who demonstrated that among patients with high myopia ranging between −5 to −30 D, the incidence of primary RRD in the 36 months after cataract surgery was 8%, compared with 1.2% among control eyes. Similarly, Al-Muammar et al^25^ demonstrated a higher incidence of primary retinal detachment after cataract extraction among patients with high myopia (2.8%) compared with nonmyopes (0.4%). Although Ripandelli et al and Al-Muammar et al highlight myopia as a significant risk factor for detachment after cataract extraction, our study suggests that its impact may be less pronounced in patients with a previous phakic RRD. This difference may stem from the specific cohort we studied, which was limited to patients who had undergone phakic RRD repair and thus differ from the general cataract surgery population.

A significant difference was observed across redetachment rates when stratified by age groups. Patients 50 to 69 years old represented the largest proportion of retinas that remained attached (66.78%) as well as retinas that redetached (57.41%), with the former being the larger proportion of the 2. By contrast, patients between 10 and 29 years old accounted for a smaller percentage of both groups (8.88% and 21.3%, respectively), with a higher proportion of patients having retinas that redetached. Moreso, the relative difference between retinas that remained attached and those that redetached was higher than that of older patients (12.5% and 9.37%, respectively). When we stratified this incidence rate by the time after cataract extraction, patients between 18 and 35 years old also consistently exhibited markedly higher redetachment rates within 1 year after surgery than all other age groups. These results may suggest that younger patients with a history of phakic RRD may be at increased risk of redetachment after cataract extractions. The shorter follow-up interval for younger patients extends this conclusion as these patients exhibited a higher incidence of detachment despite being followed for a shorter period of time postoperatively. Furthermore, when comparing the types of initial phakic retinal detachment repair surgery, younger patients were significantly overrepresented among those who had complex surgeries. This suggests that the type of initial repair surgery may contribute to the risk of redetachment among these patients.

The incidence of younger redetachments observed in our study echoes the rate of primary pseudophakic detachments reported by other studies. Daien et al^26^ found that among their youngest cohort (40-54 years), roughly 13.1% had retinas detach after cataract surgery. Such patients were at a 3.64% higher risk of detachment than older patients. Similarly, Sheu et al^27^ demonstrated that younger patients (<50 years) are at increased risk of detachment after cataract surgeries compared with older patients (>50 years), although only with an incidence of 0.5%. Lastly, Momenaei et al^28^ found that a younger age increases the odds of redetachments within 1 year after cataract surgery when patients with a history of phakic detachments and subsequent pseudophakic redetachments (mean age, 59.6 ± 8.9 years) were compared with patients without subsequent pseudophakic redetachments (mean age, 64.0 ± 9.0 years). Our results expand on these previous findings, suggesting that the observed relationship between pseudophakic detachments and patient age remains true for patients with a history of previous phakic detachments. Ultimately, our findings suggest that while myopia alone may not significantly alter the risk of retinal redetachment in patients with a history of phakic RRD, age remains a key factor. This highlights the importance of closely monitoring younger patients after cataract surgery due to their elevated risk of redetachment.

The observed relationship between age and redetachment rates may be accounted for by different pathogenetic mechanisms in younger patients. For instance, our findings suggest that complex retinal repair surgeries performed before cataract extraction may be a contributor in this population. Previous studies have reported that the incidence of redetachment ranges between 11% and 13% after combined scleral buckle/PPV; however, a significant difference has not been found when these rates are compared with those after PPV or scleral buckle alone.^29,30^ Given that combined scleral buckle/PPV are commonly reserved for complex cases, our findings may be secondary to the severity of these patients’ pathologies rather than the surgical approach itself. Additionally, the increased risk of redetachment in younger patients could be due to vitreoretinal adhesions during PVR formation, which commonly occur after intraocular surgeries and are more prevalent in younger patients.^31,32^ Alternatively, Laube et al^33^ proposed that longer axial lengths are a primary driver of pseudophakic detachments in younger patients. Younger patients may also have a greater potential for increased intraocular stress during cataract extraction due to a higher prevalence of partial PVDs.^34^ Complete PVDs, in contrast, are more prevalent in older patients and may confer some protection against detachments by reducing vitreoretinal traction.^34,35^ Lastly, the high prevalence of cataracts in younger patients may also suggest underlying pathologies, such as ocular trauma and diabetes, both of which can compromise retinal integrity during intraocular surgery.^36,37^ Comparative studies between younger and older patients within this demographic may yield more insights into other contributors to the observed relationship.

Our study had several limitations. The generalizability of our results may be limited by the selected population of eyes from the TriNetX database. White patients, those who identified as male, and those older than 50 years encompassed the largest proportion of the patients analyzed in our study, while patients from marginalized communities were disproportionately underrepresented. Myopic and highly myopic patients also represented a smaller percentage of patients than nonmyopic patients in the database. This is thought to be due to the underuse of diagnostic codes for refractive error as, more typically, most patients with RRDs are myopic. This may have subjected our statistical analysis to selective bias and limited our statistical power. For instance, given the disproportionate representation of age groups within our cohort, certain clinical factors, such as the number of comorbidities in older patients, may have exacerbated our statistical outcomes. Consequently, the true effect of these variables may have been obscured by the relatively small sample size when myopic and high-myopic patients were stratified, accounting for the nonsignificant results observed for the subgroup analysis performed across these 2 patient demographics. Additionally, ICD and CPT codes were used to extract patients from the TriNetX database that met our inclusion criteria. This may have also introduced bias due to the variability of ICD classification practices across different hospitals and our inability to fully account for laterality when filtering our data according to CPT codes.

Future studies that address these limitations should be pursued to enhance our understanding of pseudophakic redetachments. For instance, expanding the analysis with a more balanced proportion of myopes and nonmyopes may yield the true effect of these clinical factors on patient outcome. Additionally, other studies that more accurately account for laterality or those that incorporate axial lengths and refraction—variables unavailable in the TriNetX database—can also help further our understanding of how these factors affect pseudophakic redetachments. Lengthening the follow-up period can also provide insight into whether redetachments occur much later in the postoperative course after cataract extraction. Given the increased risk of redetachment among younger patients, preventative strategies and pathophysiologic mechanisms for redetachments should be explored. Future studies could evaluate the efficacy of laser retinopexy and cryopexy as prophylactic interventions against redetachments. Meanwhile, incorporating patient biomarkers, such as axial length, vitreous status, and the presence of posterior staphylomas, can shed light on which ocular characteristics contribute to the increased redetachment rates among younger patients. These studies can identify which protective measures and ocular examination findings are most pertinent when monitoring younger patients.

Our study evaluated how cataract surgeries and myopia interplay to impact a patient’s risk of redetachment. The results demonstrate that myopic status does not significantly influence the risk of redetachment after a cataract surgery in patients with a history of phakic detachments. However, our findings support the hypothesis that younger patients, particularly those aged 18 to 35 years, are at increased risk of redetachment after cataract surgery. This age group consistently exhibited higher redetachment rates across all timepoints compared with older patients, emphasizing the need for heightened vigilance and postoperative monitoring. With these results, we hope to offer insights into clinical decision-making for physicians and patients who are considering whether to undergo cataract extraction. When considering this surgery for younger patients with a history of phakic RRD, ophthalmologists should engage in thorough preoperative discussions and consider the broader clinical factors to assess the risk of redetachment beyond myopic status.

## Supplemental Material

sj-docx-1-vrd-10.1177_24741264261418517 – Supplemental material for Risk of Retinal Redetachment After Cataract Surgery Following Retinal Detachment Repair in Myopic and Highly Myopic Eyes Supplemental material, sj-docx-1-vrd-10.1177_24741264261418517 for Risk of Retinal Redetachment After Cataract Surgery Following Retinal Detachment Repair in Myopic and Highly Myopic Eyes by Yeabsira Mesfin, Leo Arnal, Anish Salvi, Karen M. Wai, Frank Brodie, Eubee Koo, Andrea L. Kossler, Euna Koo, Ehsan Rahimy, Prithvi Mruthyunjaya and Chase A. Ludwig in Journal of VitreoRetinal Diseases
