# Supplementary material for: Risk of Retinal Redetachment After Cataract Surgery Following Retinal Detachment Repair in Myopic and Highly Myopic Eyes
Source: J Vitreoretin Dis. 2026 Feb 24:24741264261418517. Online ahead of print. doi: 10.1177/24741264261418517 (PMC12932135; doi:10.1177/24741264261418517)
Supplement: sj-docx-1-vrd-10.1177_24741264261418517 – Supplemental material for Risk of Retinal Redetachment After Cataract Surgery Following Retinal Detachment Repair in Myopic and Highly Myopic Eyes [file sj-docx-1-vrd-10.1177_24741264261418517.docx]

**Supplemental Table 1.** Average follow-up intervals for each age group and results of ANOVA comparing follow-up intervals to each other.

| **Age Group (years)** | **Follow-Up Interval, mean years ± SD** | **p value** |
| --- | --- | --- |
| 20 - 29 | 8.15±7.23 | <0.05 |
| 30 - 39 | 6.58±6.75 |  |
| 40 - 49 | 6.97±6.33 |  |
| 50 - 59 | 7.43±7.54 |  |
| 60 - 69 | 8.96±8.20 |  |
| 70 - 79 | 10.14±9.38 |  |
| 80 - 89 | 8.42±10.02 |  |
